# Supplementary material for: Salt Tolerant Bacillus Strains Improve Plant Growth Traits and Regulation of Phytohormones in Wheat under Salinity Stress
Source: Plants (Basel). 2022 Oct 19;11(20):2769. doi: 10.3390/plants11202769 (PMC9608499; doi:10.3390/plants11202769)
Supplement: Supplementary file 1 [file plants-11-02769-s001.zip › plants-1955224-supplementary.pdf]

plants

MDPI

Supplementary File

Salt Tolerant Promoting *Bacillus* Strains Improves Plant Growth Traits and Regulation of Phytohormones in Wheat under Salinity Stress

Muhammad Ayaz[1], †, Qurban Ali[1], †, Qifan Jiang[1], Ruoyi Wang[1], Zhengqi Wang[1], Guangyuan Mu[2], Sabaz Ali Khan[3], Abdur Rashid Khan[1], Huijun Wu[1], Xuewen Gao[1], and Qin Gu[1]*

[1]Department of Plant Pathology, College of Plant Protection, Nanjing Agricultural University, Key Laboratory of Integrated Management of Crop Diseases and Pests, Ministry of Education, Nanjing 210095, China.
[2]Shenzhen Batian Ecological Engineering Co., Ltd, Shenzhen 518057, China.
[3]College of Environmental sciences, Biotechnology department, COMSATS, Abbottabad (22060), Pakistan.
***Correspondence:** guqin@njau.edu.cn (Q.G.); Tel: +86-025-8439-5268.
† These authors contributed equally to this study.

Table S1. Primers used in this study.

Gene Name	Primers	Sequences
	**Growth promoting genes for qPCR**	
CKX2	*CKX2* (F):	CGGGATAGGGTACAAGGAGTA
	CKX2 (R):	GTACTTGTCCTTCATCTCCACG
ERF	*ERF* (F):	TGCCCTTCGATCTGAACATG
	ERF (R):	TCCCACGAATCCTTGCTATTC
ARF	*ARF* (F):	AGATGTTCAGCACCTTCACC
	ARF (R):	CAGTCTCCATCCTTGTCTTCG
ExpA1	*ExpA1*(F):	CACCAACAAGCAGTTCTCTTA
	ExpA1 (R):	CATGGATGGACACAATACCTC
P5CS	*P5CS* (F)	GATTCTCCGATGGTGCTCGT
	P5CS (R):	TTCAACACCCACAGGTCCAC
4-HNE	*4-HNE* (F):	CAAGTACCACTTCGACCTGAG
	4-HNE (R):	CCTCTTGAACTGGATGTCGATG
ABARE	*ABARE* (F):	TTACACCGTGGAGCTTGAAG
	ABARE (R):	TTCACGTTCTCCTTGGACTG
	**Wheat salt resistance genes for qPCR**	
DREB2	*DREB1* F:	AGCAGTAATCTTCCCTGTAATG
	DREB1 R:	CAGTTGTTGGTTCACTTCTTTC

MYB	*MYB* F:	CAGATTGACGACAGCTTCTG
	MYB R:	TCTGTGACAAACTCTGCATATC
HTK1	*HTK1* F:	GGAGGATCACAAAGGTGAAG
	HTK1 R:	GCCCATCAAAGACTGAAGAA
ACTIN	*ACTIN* F:	GCCACACTGTTCCAATCTAT
	ACTIN R:	GAGCTTCTCCTTTATGTCTCTG
WRKY17	*WRKY* F:	CCTCTTTGGCTTCTCCTTTC
	WRKY R:	CAGTTGTATCCGTCCTCAAC
***Bacillus* salt-related genes for qPCR**		
DegU	*DegU* F:	TCTATCTATTCATGACGATG
	DegU R:	GTCGGAATTCATTTACTAAG
OstB	*OstB* F:	GAAGCAACAGGAGAGTATTA
	OstB R:	GCGTTGATCCTTCGAGATATT
OhrR	*OhrR* F:	GGTACGAATCCAGAGCAATTAT
	OhrR R:	AGCTTGACACCAAGCTTAAA
ComA	*ComA* F:	GAGTTGAAGGACAAGTAGCC
	ComA R:	GAATCGCAGAGGTCATCATC
SodA	*SodA* F:	GGTCAAATGCTTCGTCTTATTG
	SodA R:	AGAGCTAGAAGAGGGATGAC
OpuAC	*OpuAC* F:	ATACATATACAGGCATTCAG
	OpuAC R:	CAGAATCAGACTTCCTAATC

**Primers used for salt stress-related genes detections in *Bacillus* strains**		
O*puAC*	*OpuAC* F:	GACCGCCCTGCTTGTCTTTT
	OpuAC R:	CTAGACCCATGCGAAGACCT
OstB	*OstB* F:	CTGTCTGAATGGCAGACGAA
	OstB R:	CGCAACGTAAATCCCTTCAT
DegU	*DegU* F:	TCACCAATTATTCCGTGAAGG
	DegU R:	TCATTTCTACCCAGCCGTTT
ComA	*ComA* F:	GCTTGATCCGATGATTCTCC
	ComA R:	CGACACCGCCTACTTCACTT
OhrR	*OhrR* F:	ATGGCAGAGCGTTTTGTAGG
	OhrR R:	GGCAAAGTCCACCAGTTTGT
SODA	*SODA* F:	AAATTACCTCCGCTCCCTTAC
	SODA R:	AAGTAGAAGGTGTTGGCTGG
